# Multi-dimensional composite catalyst NiFeCoMoS/NFF for overall electrochemical water splitting†

**DOI:** 10.1039/d4ra08605h

**Published:** 2025-02-17

**Authors:** Zhaojun Tan, Shuaihui Guo, Wen Wang, Gang Li, Zhenwei Yan

**Affiliations:** a School of Mechanical Engineering, North China University of Water Resources and Electric Power Zhengzhou 450045 PR China 13603990078@163.com yanzhenwei@163.com +86 13603990078 +86 18638513931

## Abstract

Precise catalyst design is essential in the electrolysis of water to deliver clean energy, where the challenge is to construct highly active sites at the electrocatalyst interface. In this study, CoPVP/NFF (NiFe foam) and Mo–CoPVP/NFF precursors were synthesized sequentially in a hydrothermal procedure using NiFe foam as substrate with the ultimate formation of a NiFeCoMoS/NFF electrocatalyst by vulcanization at 350°. The NiFeCoMoS/NFF system exhibits a complex 1D–2D–3D composite structure with 1D nanoparticles attached to a 2D nano-paper on the surface of the 3D NiFe foam. The overpotentials associated with hydrogen and oxygen evolution by NiFeCoMoS/NFF are 123 mV and 245 mV, respectively, at a current density of 10 mA cm^−2^. A three-electrode system using NiFeCoMoS/NFF as working and counter electrode has been assembled that can generate current densities of 100 mA cm^−2^ at voltages of 1.87 V. Theoretical (DFT) calculations have shown that NiFeCoMoS/NFF exhibits favorable H adsorption energetics and a low OER reaction barrier. This study has identified a viable means of enhancing the efficiency of water electrolysis by regulating catalyst surface structure.

## Abstract 2

A complex 1D–2D–3D composite structure (NiFeCoMoS/NFF) was prepared by vulcanizing the CoPVP/NFF (NiFe foam) and Mo–CoPVP/NFF precursors synthesized sequentially in a hydrothermal procedure using NiFe foam as substrate.

## Graphical abstract

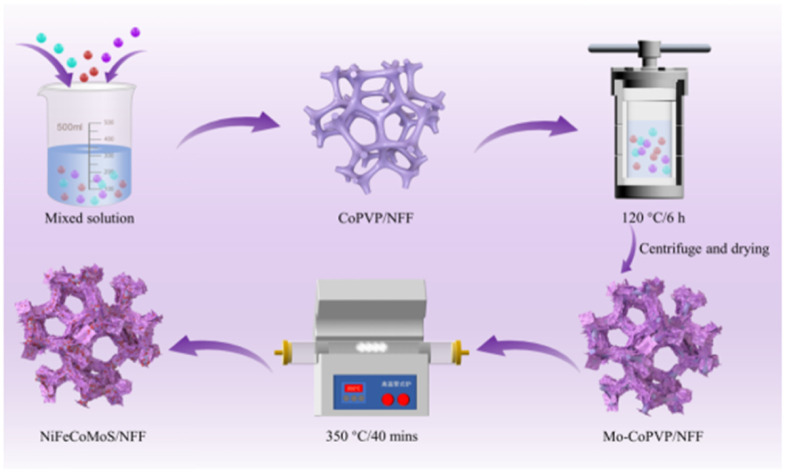

## 1. Introduction

The generation of clean energy from water splitting is viewed as a viable solution that can address current energy demands. Water splitting serves as a sustainable means of hydrogen generation and involves both hydrogen evolution reaction (HER) and oxygen evolution reactions (OER).^1–3^ In general, precious metals are employed as effective catalysts in water splitting, notably Pt as an electrocatalyst in HER and RuO_2_ in OER.^4–6^ However, low reserves and high costs have limited precious metal utilization.^7–9^ Alternative non-precious catalysts, including oxides, hydroxides, phosphides, nitrides and sulfides have been developed to promote HER, OER and overall water splitting.^10–14^

Taking an overview of the non-precious metals used in electrocatalytic HER and OER, molybdenum, nickel, iron and cobalt sulfides show significant promise.^15–18^ In order to improve the performance of these catalysts, various strategies such as morphology, defect and heterostructure engineering have been considered.^19–22^ The nanostructure of electrocatalysts can be modified to a certain degree by controlling synthesis conditions such as temperature, concentration, and surfactant. In a structure sensitive catalytic process, different nanostructures exhibit distinct properties that can be employed to effectively optimize electrocatalytic performance, as in the case of MoO_3_ nanodots on MoS_2_ nano-paper, MoNi_4_ nanosheets attached to MoO_3−*x*_ nanorods, and NiS_2_/N–NiMoO_4_ nanosheets/nanowires,^23–25^ which have been developed for use in water electrolysis. Different transition metal electrocatalysts may be combined to form heterojunctions, where the coupling interfaces at the heterojunction in tandem with synergistic effects involving different active components can modulate catalyst electron transfer efficiency and the number and nature of the active sites. A significant range of heterojunction catalysts^26–33^ have been designed to enhance water electrolysis efficiency, such as MoS_2_/Fe–NiCo_2_O_4_, Co-LDH@ZIF-67, MoS_2_/MoP, LiNiO_2_/NiOOH, MoS_2_/WS_2_, and MoO_2_–FeP@C. Sulfur-based heterojunctions, such as CoS-doped β-Co(OH)_2_/MoS_2_, MoS_2_/Fe_5_Ni_4_S_8_, MoS_2_/Ni_3_S_2_, NiS_2_/MoS_2_, MoS_2_/Co_9_S_8_/Ni_3_S_2_/Ni, and MoS_2_/(Co,Fe,Ni)_9_S_8_ have been systematically studied for the purpose of enhancing the rate of water electrolysis.^34–41^ With respect to transition metal dihalides,^42^ MoS_2_ and Ni_3_S_2_ have been employed as HER electrocatalysts, but the HER performance of transition metal sulfides is limited by a low charge transport efficiency and high contact resistance with the substrate material. It should be noted that S–H bond formation on the catalyst surface facilitates the adsorption of H (H_ads_), but the energy barrier to convert H_ads_ to free H is high. Moreover, the OER performance of the metal sulfide is insufficient for practical application. Given the limitations of current catalyst formulations, it is necessary to integrate morphology engineering and heterojunction engineering in order to optimize adsorption energetics and improve water electrolysis efficiency.

In this study, Mo–CoPVP/NFF precursors have been synthesized using a hydrothermal procedure with NiFe foam (NFF) as substrate, and the NiFeCoMoS/NFF water splitting electrocatalyst was obtained by vulcanization at 350 °C. The NiFeCoMoS/NFF system presents a multi-dimensional composite structure: 1D nanoparticles are formed on twisted 2D nano-papers covering the surface of the 3D NiFe foam. The NiFeCoMoS/NFF catalyst exhibits excellent electrocatalytic activity with overpotentials of 123 mV for HER and 245 mV for OER at current densities of 10 mA cm^−2^. The remarkable electrocatalytic performance of this polymetallic sulfide electrocatalyst is attributed to the simultaneous modulation of active ingredients and geometric structure, optimization of charge transfer efficiency, an abundance of electrocatalytic active sites and synergistic effects at the heterojunction interfaces. The results of Density Functional Theory (DFT) calculations have shown that the CoS/MoS heterojunction offers an optimal H adsorption energy and the NiFeS species lower the reaction energy barrier for OER. A two-electrode NiFeCoMoS/NFF assembly can deliver a stable current density of 100 mA cm^−2^ at voltages of 1.87 V. The results generated have established the viability and enormous potential of this catalytic system in electrocatalytic water splitting.

## 2. Experimental procedures

All the reagents, including Ni foam, Fe foam and NiFe foam (Lizhiyuan Co.), cobalt acetate (Sinopharm Chemicals), polyvinylpyrrolidone (PVP Aladdin), Pt/C catalyst (10 wt%, Sinopharm Chemicals), RuO_2_ catalysts (10 wt%, Sinero Technology Co.), ammonium molybdate tetrahydrate (Sinopharm Chemicals), sulfur powder (Sinopharm Chemicals), KOH (Aladdin), ethanol (Aladdin), and Nafion (5 wt%, DuPont) were purchased from commercial suppliers and used without further purification. Milli-Q water (18 MΩ cm) was used in all the experiments.

### 2.1. Materials

#### NiFeCoMoS/NFF

(1) A section (1 × 3 cm) of Ni–Fe foam (NFF) was rinsed with 3 M HCl solution, acetone, anhydrous ethanol and deionized water for 10 minutes, respectively, and vacuum dried at 60 °C for 3 h. (2) A sample (0.18 g) of cobalt acetate and 250 mg (8000 W) PVP were dissolved in 30 mL absolute ethanol, the solution was stirred for 20 minutes and transferred to a 50 mL PTFE reactor. The dried NFF was added and the CoPVP/NFF precursor obtained after a hydrothermal reaction at 120 °C for 6 h. (3) Ammonium molybdate tetrahydrate (0.53 g) was dissolved in 35 mL deionized water, the CoPVP/NFF precursor added to the solution, and Mo–CoPVP/NFF was produced in a hydrothermal reaction at 180 °C for 4 h. (4) Sulfur powder (1 g) and the Mo–CoPVP/NFF array were placed upstream and downstream of a quartz tube, respectively. The quartz tube was heated to 350 °C for 40 minutes in Ar with the resultant formation of the NiFeCoMoS/NFF electrocatalyst.

#### NiCoMoS/NF (Ni foam) and FeCoMoS/FF (Fe foam)

The synthesis followed the procedure for NiFeCoMoS/NFF, replacing the Ni–Fe foam with Ni foam and Fe foam, respectively.

#### NiFeMoS/NFF and NiFeCoS/NFF

The synthesis followed the procedure for NiFeCoMoS/NFF, but no cobalt acetate or ammonium molybdate was included in the hydrothermal reaction, respectively.

NiFeS was obtained by directly vulcanization of Ni–Fe foam.

#### Pt–C/NFF and RuO_2_/NFF

A sample (20 mg) of 10% Pt–C powder, 500 μL anhydrous ethanol, 500 μL deionized water and 50 μL 5% Nafion solution were mixed and sonicated for 30 min; the Pt–C/NFF was obtained by dropwise addition of the mixture onto NFF. The preparation of RuO_2_/NFF followed the same procedure.

### 2.2. Structural characterization

#### X-ray diffraction

The crystal structure of the electrocatalysts was determined using a Smart Lab X-ray diffractometer (Riki Co. Ltd, Japan) with a Cu-Kα radiation source (*λ* = 0.15406 nm), 40 kV voltage, 30 mA current, a scanning speed of 5° min^−1^ and scanning range of 10°–80°.

#### Scanning electron microscopy (SEM)

Samples were analyzed using a Quanta 400 FEG field emission scanning electron microscope (FEI, USA), equipped with an EDS energy spectrometer used to determine surface elemental distribution. Self-supporting electrocatalyst samples were directly adhered to the sample carrier using conductive adhesive and analyzed at an acceleration voltage of 20 kV.

#### Transmission electron microscopy (TEM)

The samples were examined using a Tecnai G2 F20 S-Twin field emission transmission electron microscope (FEI) at an accelerating voltage of 200 kV.

#### X-ray photoelectron spectroscopy (XPS)

The samples were scanned using an ESCALAB 250X1 photoelectron spectrometer (Thermo Scientific) with A1 Kα (*λ* = 1486.6 eV) as the radiation source.

### 2.3. Electrochemical measurements

The CHI 760E electrochemical workstation system with a three-electrode system was used to measure electrocatalytic performance in a 1.0 M KOH (pH = 13.71) solution. A platinum sheet was utilized as counter electrode, the catalyst was the working electrode, and Hg/HgO served as reference electrode. The electrode potential, *E* (Hg/HgO), was converted to *E* (RHE) according to the equation, *E* (RHE) = *E* (Hg/HgO) + 0.098 + 0.0591 × pH. The sweep speed of the linear scan voltammogram (LSV) was 2 mV s^−1^. In the application of electrochemical impedance spectroscopy (EIS), the open circuit potential was first surveyed with input as the reference potential; the frequency range was 0.1–10000 Hz at an amplitude of 5 mV. Chronoamperometric (CP) tests were performed to evaluate the stability of the electrocatalysts at a current density of 10 mA cm^−2^. When testing the electrochemically active surface area (ECSA) of the electrocatalysts, the potential interval was 20 mV for HER and for OER. The experimental measurements were not compensated by iR.

### 2.4. Density functional theory (DFT) calculations

The calculations were carried out using the CASTEP module of Material Studio. The ultra-soft pseudopotential and generalized gradient approximation proposed by Perdew, Burke, and Ernzerhof (GGA-PBE) was adopted, and the cutoff energy of the planewave basis was set to 571 eV. The Monkhorst–Pack method was employed for *k*-space sampling with a grid size of 1 × 1 × 1. The convergence criteria for energy and force were 2.0 × 10^−5^ eV per atom and 0.05 eV Å^−1^, respectively. The vacuum region along the *z* axis was 12 Å so that the interactions between adjacent models can be neglected. The Gibbs free energy (Δ*G*_H_) for H* on the surface of the electrocatalysts was obtained using the method formulated by Norskov:Δ*G*_H_ = *E*_surf-H_ − *E*_surf_ − 0.5*E*_H_2__ − *T*Δ*S*where *E*_surf-H_ represented the energy of the H adsorbed surface, *E*_surf_ represented the energy of the surface, *E*_H_2__ represented the energy of H_2_, and *T*Δ*S* represented the entropy change.

The OER process occur through the following four electrons steps:surf + H_2_O = surf-OH + H^+^ + e^−^surf-OH = surf-O + H^+^ + e^−^surf-O + H_2_O = surf-OOH + H^+^ + e^−^surf-OOH = surf + O_2_ + H^+^ + e^−^

So Δ*G* can be calculated as:Δ*G*_1_ = *E*_surf-OH_ + *E*_H_2_/2_ + eU − *E*_surf_ − *E*_H_2_O_Δ*G*_2_ = *E*_surf-O_ + *E*_H_2_/2_ + eU − *E*_surf-OH_Δ*G*_3_ = *E*_surf-OOH_ + *E*_H_2_/2_ + eU − *E*_surf-O_ − *E*_H_2_O_Δ*G*_4_ = *E*_surf-O_2__ + *E*_surf_ + *E*_H_2_/2_ + eU − *E*_surf-OOH_

## 3. Results

### 3.1. Synthesis and characterization

**Fig. 1:**
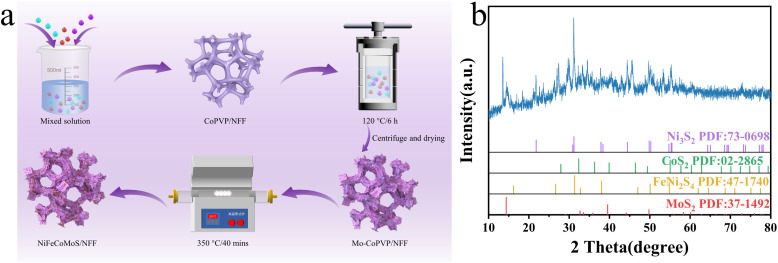
(a) Schematic representation of the catalyst synthetic route; (b) XRD pattern of NiFeCoMoS/NFF.

The synthesis procedure for NiFeCoMoS/NFF is shown in Fig. 1(a), where the CoPVP/NFF precursor and Mo–CoPVP/NFF were prepared by hydrothermal treatment using NiFe foam (NFF) as the substrate. The self-supported NiFeCoMoS/NFF was obtained following vulcanization of Mo–CoPVP/NFF at 350° in Ar.

The catalyst was analyzed by XRD to determine sample crystallinity. The XRD pattern for the NiFeCoMoS/NFF powder (Fig. 1(b)) presents peaks at 31.1°, 49.8° and 21.8° that are attributed to the [1 −1 0], [2 1 0], and [1 0 0 ] crystal planes of Ni_3_S_2_ [JCPDS No. 73-0698]. The peaks at 32.3°, 55.1° and 39.9° are ascribed to the [2 0 0], [3 1 1] and [2 1 1] crystal planes of CoS_2_ [02-2865], the peaks at 31.3°, 38° and 54.8° are due to the [3 1 1 ], [4 0 0 ] and [4 4 0] crystal planes of Fe(NiS_2_)_2_ [47-1740], and the peaks at 14.3, 39.5 and 49.7 are due to the [0 0 2 ], [1 0 3] and [1 0 5] crystal planes of MoS_2_ [37-1492]. The NiFeCoMoS/NFF XRD pattern confirms the successful synthesis of the polymetallic sulfide catalyst. It should be noted that the NiFe foam was not completely converted to sulfide, and the remaining NiFe component served as a structural skeleton that can enhance catalyst conductivity.

**Fig. 2:**
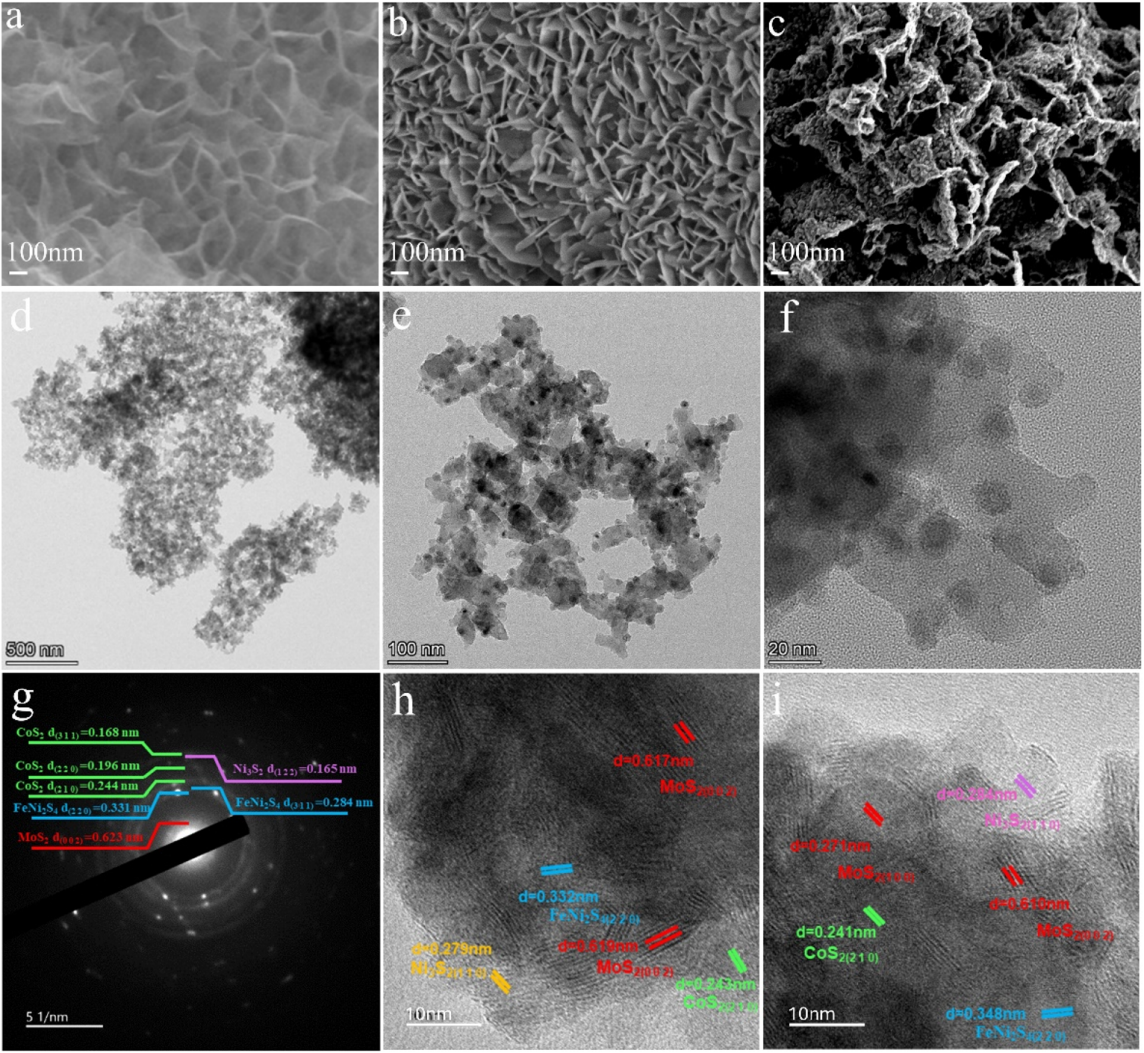
SEM images of (a) CoPVP/NFF, (b) Mo–CoPVP/NFF; (c) NiFeCoMoS/NFF; (d)–(f) TEM images of NiFeCoMoS/NFF; (g) SAED image of NiFeCoMoS/NFF; (h) and (i) HRTEM image of NiFeCoMoS/NFF.

Catalyst morphology was assessed by scanning electron microscopy (SEM), and representative images are shown in Fig. 2. The CoPVP/NFF (Fig. 2(a)) precursor is characterized by CoPVP 2D smooth nanosheets that are evenly distributed on the surface of the 3D NiFe foam. The Mo–CoPVP/NFF sample also exhibits a smooth 2D nanosheet arrangement (Fig. 2(b)) where sample morphology has not been significantly altered due to the inclusion of Mo. In contrast, NiFeCoMoS/NFF (Fig. 2(c)) exhibits a rough or twisted surface that supports a number of 1D particles. These particles contribute to the surface roughness and can increase the active surface area and contribute to catalyst performance. The SEM analysis suggests that the inclusion of the Mo dopant does not significantly alter sample micromorphology, but the vulcanization process disrupted and distorted the nanosheets, and generated 1D particles on the surface. The EDS mapping of NiFeCoMoS/NFF (ESI Fig. 1†) has established an even distribution of Ni, Fe, Co, Mo, and S on the catalyst surface.

**Fig. 3:**
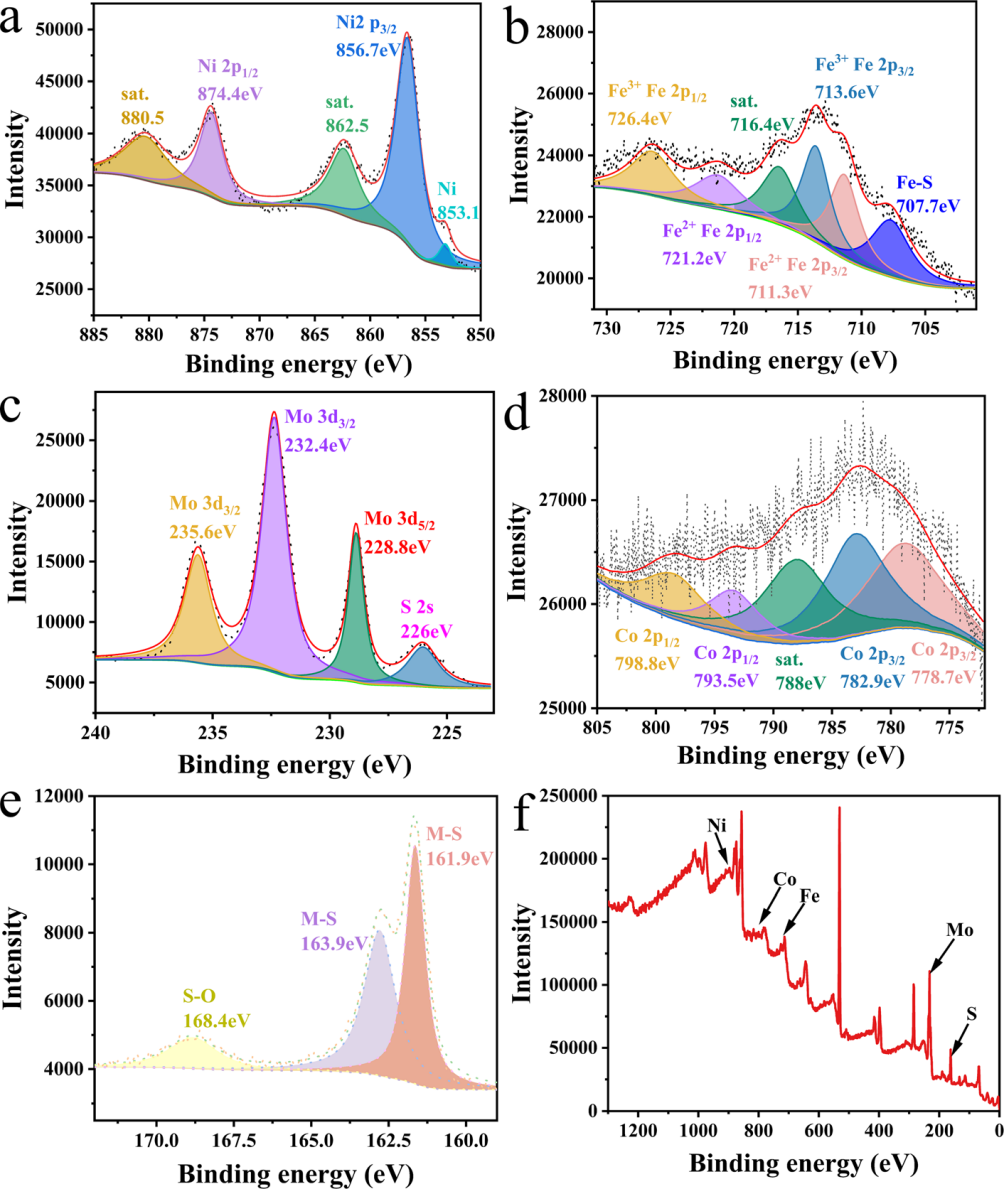
High-resolution XPS spectra for (a) Ni 2p, (b) Fe 2p, (c) Mo 3d, (d) Co 2p, (e) S 2p, and (f) XPS survey spectra of NiFeCoMoS/NFF.

The surface features were further evaluated using transmission electron microscopy (TEM), and representative images are presented in Fig. 2(d)–(i). An overall irregular and granular appearance is evident (Fig. 2(d)). At higher magnification, small particles (Fig. 3(e) and (f)) can be observed associated with the layered structure, suggesting the occurrence of nanoparticles embedded in the 2D nanosheets. In this composite Ni, Fe, Co, and Mo quaternary sulfide structure, the preparation pathway should result in the formation of inner Ni and Fe sulfides with outer Co and Mo sulfide layers. A Ni and Fe component may react with excess S to form granular Ni and Fe sulfides with lower surface energy that migrate to the surface of the layered Co and Mo sulfides. The results of SAED analysis (Fig. 3(d)) and HRTEM lattice constant measurements (Fig. 2(g)–(i)) have established the presence of CoS_2_ (311) (220) (210), MoS2 (002) (100), Ni_3_S_2_ (122) (110), and FeNi_2_S_4_ (220) crystal planes. These results are consistent with the XRD analysis.

The SEM and TEM measurements illustrate that the vulcanization step distorted the nanosheets and created 1D nano-particles on the surface of the resultant nanosheets. The components (MoS_2_, Ni_3_S_2_, CoS_2_, and FeNi_2_S_4_) are distributed to generate a multi-heterojunction catalyst showing a 1D–2D–3D multi-dimensional composite morphology.

The catalyst surface chemical state was determined by XPS analysis; the results are presented in Fig. 3. The Ni 2p spectrum (Fig. 3(a)) exhibits a minor peak at 853.1 eV that can be indexed to metallic nickel associated with the NFF substrate. The main peaks at 856.7 eV (Ni 2p_3/2_) and 874.4 eV (Ni 2p_1/2_) can be attributed to Ni_3_S_2_/NiFeS. The Ni 2p spectrum of Ni_3_S_2_ (ESI Fig. 2†) is characterized by peaks at 856.1 eV and 873.7 eV,^43–45^ indicating that the Fe dopant served to shift the Ni 2p XPS signal. This response can be explained by the lower electronegativity of Fe relative to Ni, resulting in an increase in electron density around Ni atoms and a consequent negative shift in binding energy. The modulation of the Ni electron cloud density due to the inclusion of Fe atoms is conducive to OER, which can be confirmed by electrochemical measurements and density functional simulations.

The Fe 2p XPS spectrum (Fig. 3(b)) exhibits a satellite peak at 716.4 eV where the peak at 707.7 eV can be assigned to an Fe–S bond, the peak at 711.3 eV is due to the Fe 2p_3/2_ and Fe 2p_1/2_ components of Fe^2+^ species, and peaks at 713.6 and 726.4 eV may be attributed to the Fe 2p_3/2_ and Fe 2p_1/2_ components of Fe^3+^ species.^46–48^ The XPS response is consistent with a NiFeS phase.

As shown in Fig. 3(c), the XPS peak at 226 eV can be assigned to the S 2s orbital, and the peaks at 228.8 eV (Mo 3d_5/2_) and 232.3 eV (Mo 3d_3/2_) are attributed to MoS_2_ species associated with NiFeCoMoS/NFF. The peak at 235.65 eV (Mo 3d_3/2_) suggests some degree of Mo oxidation in air. The XPS spectrum for MoS_2_ (ESI Fig. 3†) is characterized by a Mo 3d_3/2_ peak at 231.9 eV, which is shifted to a higher binding energy in NiFeCoMoS/NFF,^49,50^ suggesting the electron density around the Mo atom is lowered which may enhance HER performance.

The Co 2p XPS spectrum is presented in Fig. 3(d) where peaks at 778.7 eV (Co 2p_3/2_), 793.5 eV (Co 2p_1/2_), 782.9 eV (Co 2p_3/2_), and 798.8 eV (Co 2p_1/2_) are due to Co^3+^ species associated with CoS_2_, with a satellite peak at 788 eV.^51–53^ In the case of the S 2p spectrum (Fig. 3(e)), the peaks at 161.6 eV, 163.9 eV and 168.4 eV can be attributed to a metal–S bond, suggesting partial oxidation in air.^54–56^ The XPS analysis has indicated that the active components in NiFeCoMoS/NFF include Ni_3_S_2_, NiFeS, MoS_2_ and CoS_2_, which form heterojunctions, as suggested by the TEM measurements.

### 3.2. Electrocatalytic performance

**Fig. 4:**
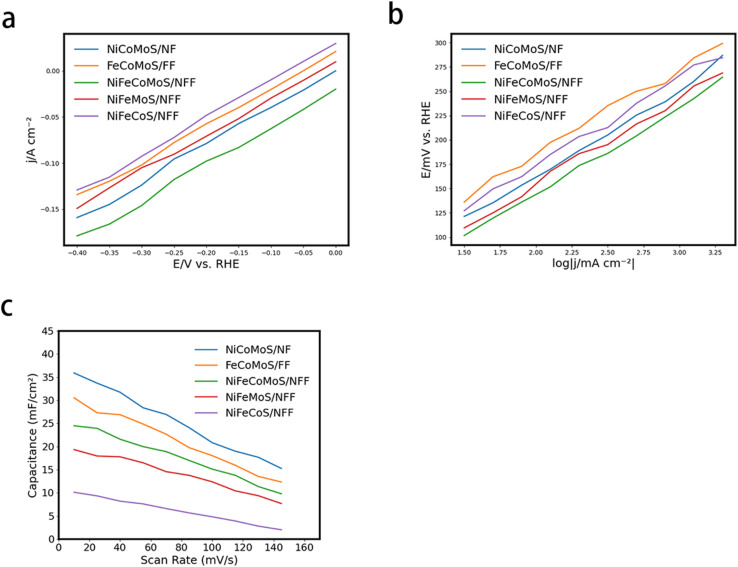
(a) Polarization curves for NiCoMoS/NF, FeCoMoS/FF, NiFeCoMoS/NFF, NiFeMoS/NFF, and NiFeCoS/NFF; (b) Tafel plots for NiCoMoS/NF, FeCoMoS/FF, NiFeCoMoS/NFF, NiFeMoS/NFF, and NiFeCoS/NFF: (c) Cdl curves associated with HER.

Fig. 4(a) shows a steeper increase in current density at lower potentials, indicating that NiFeCoMoS/NFF exhibits higher electrocatalytic activity for the hydrogen evolution reaction (HER) compared to the other materials. This suggests that it can efficiently drive the HER at lower overpotentials. In Fig. 4(b), the Tafel plot reveals a smaller slope, indicating that NiFeCoMoS/NFF follows a more kinetically favorable reaction pathway, which implies faster electron transfer and enhanced HER kinetics. Fig. 4(c) demonstrates relatively higher capacitance values, which correspond to a larger electrochemical surface area (ECSA) and more active sites for the HER, enhancing the material's overall catalytic efficiency.

In summary, NiFeCoMoS/NFF outperforms the other four materials in terms of electrocatalytic activity, reaction kinetics, and active-site availability for HER.

The synergism associated with the 1D–2D–3D composite morphology which is responsible for improved charge transfer efficiency associated with the well-dispersed active sites in NiFeCoMoS/NFF results in enhanced HER performance. As the HER performance of FeCoMoS/NF, FeCoMoS/FF and NiFeCoMoS/NFF are similar (ESI Fig. 5†), it is reasonable to infer that the main active component that determines HER performance is the CoMoS heterojunction.

**Fig. 5:**
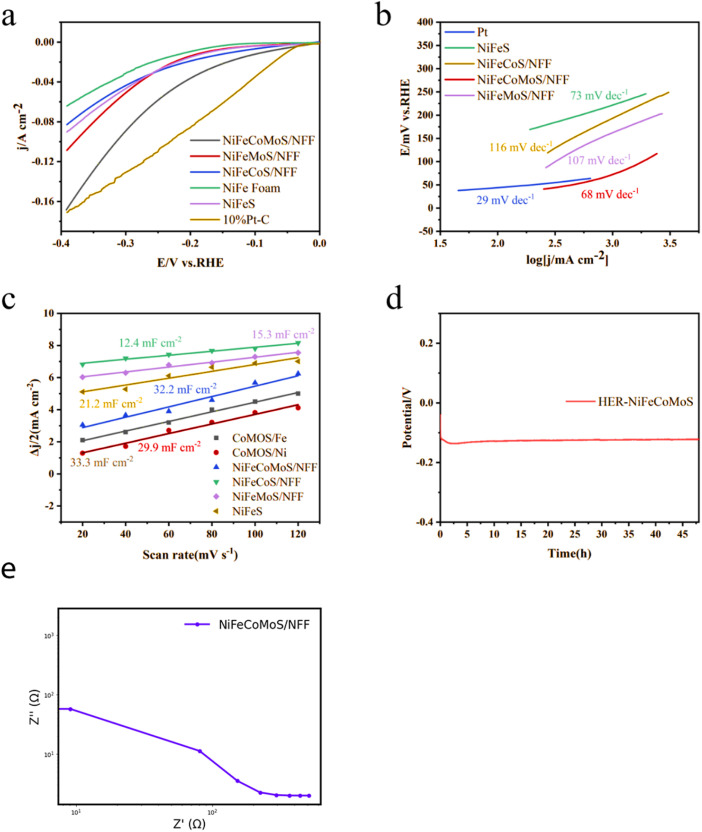
(a) Polarization curves associated with HER; (b) Tafel plots for Pt/C, NiFeS, NiFeMoS/NFF, NiFeCoS/NFF, and NiFeCoMoS/NFF: (c) Cdl curves associated with HER; (d) chronopotentiometry curve for NiFeCoMoS/NFF; (e) EIS spectrum of NiFeCoMoS/NFF.

Catalytic hydrogen evolution performance was evaluated by linear scanning voltammetry (LSV) at 25 °C in 1 mol L^−1^ KOH solution using a three-electrode system. The polarization curves for NiFeMoS/NFF, NiFeCoS/NFF, NiFeCoMoS/NFF, NiFeS, 10% Pt–C/NFF, and NFF are shown in Fig. 5(a). When compared with NiFeMoS/NFF (176 mV and 316 mV), NiFeCoS/NFF (137 mV and 343 mV) and NiFeS (175 mV and 331 mV), NiFeCoMoS/NFF required a lower overpotential (123 mV and 252 mV) to deliver current densities of 10 and 60 mA cm^−2^. Moreover, a relatively low overpotential (314 mV) was required in the case of NiFeCoMoS/NFF at a current density of 100 mA cm^−2^. It should be noted that at an overpotential of 390 mV, NiFeCoMoS/NFF and 10% Pt–C/NFF delivered a current density of 167 and 190 mA cm^−2^, respectively, and the electrocatalytic activity of NiFeCoMoS/NFF was 12% lower than 10% Pt–C/NFF. The HER kinetics were assessed using Tafel plots (Fig. 5(b)). From a consideration of the slopes associated with NiFeMoS/NFF (107 mV dec^−1^), NiFeCoS/NFF (116 mV dec^−1^), and NiFeS (73 mV dec^−1^), NiFeCoMoS/NFF is characterized by the lowest value (68 mV dec^−1^), indicating faster HER kinetics.

The electrochemical active surface area (ECSA) is an important parameter that determines electrode performance, where the value is proportional to the double-layer capacitance (Cdl), which is presented in Fig. 5(c). The highest Cdl value associated with NiFeCoMoS/NFF implies the largest ECSA (Fig. 5 and ESI Table 1†), indicating that the NiFeCoMoS/NFF catalyst has higher intrinsic activity.

Stability is a key factor in the practical application of a new catalyst. The chronoamperometric curve for NiFeCoMoS/NFF, presented in Fig. 5(d), shows no obvious variation in overpotential over an operating period in excess of 45 hours, demonstrating HER stability.

### 3.3. Electrochemical impedance spectroscopy (EIS) analysis

To understand the physical processes of the NiFeCoMoS/NFF catalyst during water-splitting, electrochemical impedance spectroscopy (EIS) analysis was performed. Fig. 5(e) shows a small charge-transfer resistance (*R*_ct_) in the high-frequency region, indicating excellent charge-transfer ability. Compared to other catalysts, NiFeCoMoS/NFF has a significantly lower *R*_ct_, confirming its rapid charge-transfer properties and high efficiency in electron transfer. The EIS analysis also shows stable electrochemical performance throughout the reaction, further supporting its catalytic stability. NiFeCoMoS/NFF outperforms other catalysts in impedance, demonstrating higher electron conductivity and stronger catalytic activity.

In summary, EIS analysis confirms the superior charge-transfer performance and catalytic efficiency of NiFeCoMoS/NFF for both HER and OER.

**Fig. 6:**
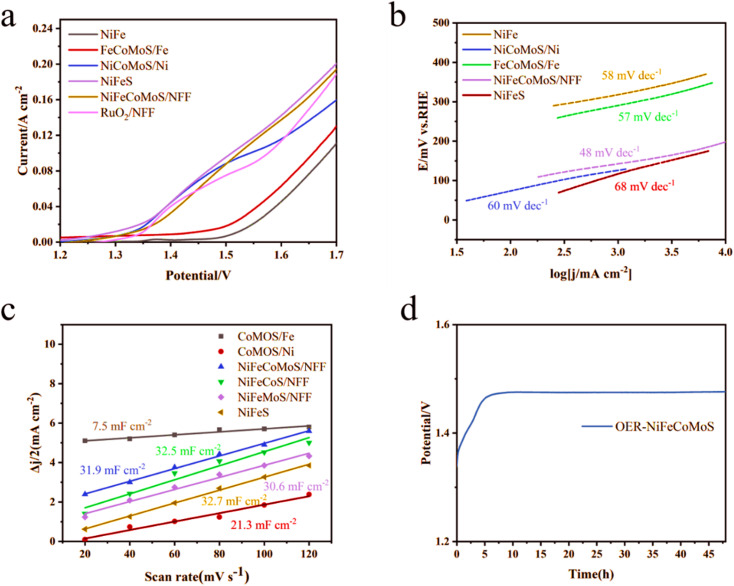
(a) Polarization curves associated with OER; (b) Tafel plots for NiFe, NiCoMoS/Ni, FeCoMoS/Fe, NiFeCoMoS/NFF, and NiFeS: (c) Cdl curves associated with OER; (d) chronopotentiometry curve for NiFeCoMoS/NFF.

The overall efficiency of the water electrolysis process is governed by the OER rate. The associated polarization curves are presented in Fig. 6(a). In this system, NiFeCoMoS/NFF exhibits a high OER rate, requiring an overpotential of 245 mV to deliver a current density of 10 mA cm^−2^, lower than NiCoMoS/Ni (268 mV) and FeCoMoS/Fe (320 mV). When the potential is 1.7 V, NiFeCoMoS/NFF, NiCoMoS/Ni and FeCoMoS/Fe deliver a current density of 195 mA cm^−2^, 162 mA cm^−2^ and 136 mA cm^−2^, respectively. The calculated Tafel slope offers some insight into the OER kinetics mechanism. As shown in Fig. 6(b), the slope for NiFeCoMoS/NFF (48 mV dec^−1^) suggests a faster OER rate compared with NiCoMoS/Ni (60 mV dec^−1^) and FeCoMoS/Fe (57 mV dec^−1^). Moreover, the Cdl (Fig. 6(c) and ECSA (797.5 cm^2^, ESI Table 2†) for NiFeCoMoS/NFF) was higher than NiCoMoS/NF (532.5 cm^2^) and FeCoMoS/FF (187.5 cm^2^), suggesting the generation of abundant active sites in NiFeCoMoS/NFF as a result of the geometric morphology. In terms of stability, the overpotential of NiFeCoMoS/NFF at a current density of 10 mA cm^−2^ was essentially invariant following an initial induction period and showed little variation after 50 hours of reaction, as shown in Fig. 6(d). The OER activity for NiFeCoMoS/NFF, NiFeS, NiFeCoS/NFF and NiFeMoS/NFF essentially converged (ESI Fig. 6†), suggesting that NiFeS is the principal active component that determines OER performance.

Given the enhanced HER and OER performance, NiFeCoMoS/NFF were incorporated in the cathodes and anodes of two-electrode systems. The resultant NiFeCoMoS/NFF||NiFeCoMoS/NFF electrodes achieved robust catalytic performance, requiring voltages of 1.598 and 1.87 at 10 mA cm^−2^ and 100 mA cm^−2^ in 1 M KOH at 25 °C. In comparison of NiCo_2_S_4_, δ-FeOOH, MoS_2_/NiS_2_, Ni_3_S_2_–CoMoS_*x*_, Co–Fe–NiSe_2_, MoS_2_/NiS_2_, MoS_2_/NiS and so on, the inferior voltage at 10 mA cm^−2^ is gained for NiFeCoMoS/NFF (ESI Table 3†).

### 3.4. First-principles calculations

**Fig. 7:**
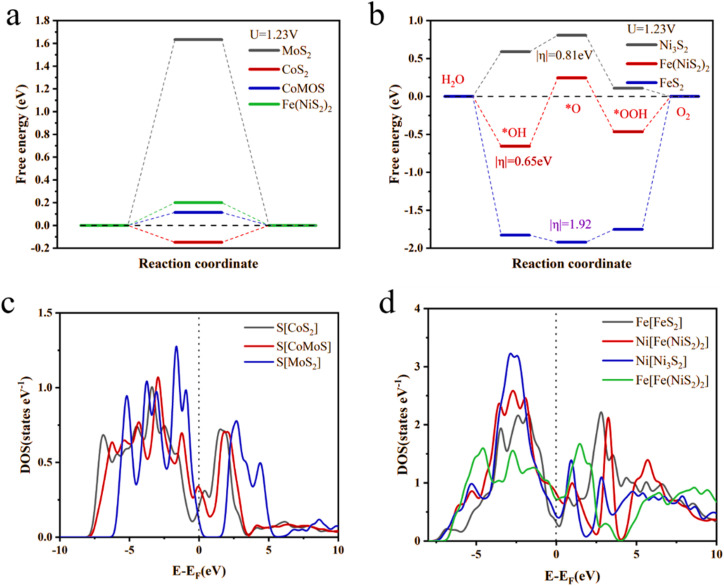
Free energy diagrams for (a) HER and (b) OER; DOS plots of S, Ni and Fe atom associated with (c) HER and (d) OER.

In order to explore the relationship between catalytic activity and NiFeCoMoS/NFF structure, the variation in Gibbs free energy was calculated for each step in the HER and OER reactions using the Material Studio CASTEP module (ESI Fig. 7†). The hydrogen adsorption energy (Δ*G*_H*_) is recognized as a key indicator in evaluating HER performance. As shown in Fig. 7(a), relative to MoS_2_ (Δ*G*_H*_ = 1.62 eV), NiFeS (Δ*G*_H*_ = 0.22 eV) and CoS_2_ (Δ*G*_H*_ = −0.18 eV), Δ*G*_H*_for the sulfur sites associated with the CoMoS (Δ*G*_H*_ = 0.14 eV) heterojunction is closer to zero, indicating a more favorable H interaction that significantly reduces the thermodynamic energy barrier for hydrogen production. The results of the first-principles calculations are consistent with the LSV measurements, supporting the predominant role of the CoMoS heterojunction in promoting the HER reaction.

The oxidation of water in an alkaline solution involves four synergistic proton-electron transfer steps, and the associated free energy profiles are presented in Fig. 7(b). It is clear that the potential rate-determining step (PDS) using NiFeCoMoS/NFF is the electrochemical conversion of H_2_O to *OH with an associated energy barrier of 0.65 eV, lower than Ni_3_S_2_ (0.81 eV) and FeS_2_ (1.92 eV). The data suggest that NiFeS can have an effect on lowering the overpotential and is indispensable in stabilizing the intermediates and driving the electrochemical kinetics.

In order to gain insight into the electronic structure of the catalytic surface, the density of state (DOS) of the surface atoms has been calculated. As shown in Fig. 7(c), the DOS of the S atom on the surface of the CoMoS heterojunction near the Fermi level is higher than that of MoS_2_ and CoS_2_, suggesting an enhanced conductivity of the CoMoS heterojunction, which facilitates electron transport in HER. The DOS values of Ni and Fe atoms near the Fermi level in the case of Fe(NiS_2_)_2_ were higher than Ni_3_S_2_ and FeS_2_, indicating that the Ni and Fe atoms associated with the Fe(NiS_2_)_2_ surface possess higher activity. Both the simulations and experimental measurements suggest improved OER and HER as a result of the synergistic effects of oxidation/hydrogenation-induced surface reconfiguration.

The enhanced electrocatalytic activity is firstly attributed to the 3D composite structure of NiFeCoMoS/NFF, which ensures good mass transport and gas permeability. Secondly, the interfaces in this heterogeneous composite structure involve a synergism of active components that promotes and enhances charge transfer efficiency. Thirdly, the distorted or twisted 2D nanosheets and associated 1D nanoparticles contribute to improved conductivity and increased active sites, as confirmed by the EIS and ECSA measurements. Finally, the MoS/CoS heterojunction accelerates the HER process, where surface NiFeS species play a critical role in OER. These NiFeCoMoS/NFF structural features combine to deliver a highly efficient water splitting performance that can contribute to the development of practical water electrolysis materials.

## 4. Conclusions

The CoPVP/NFF and Mo–CoPVP/NFF precursors were prepared by sequential hydrothermal treatment using NiFe foam (NFF) as substrate. Vulcanization of Mo–CoPVP/NFF at 350 °C generated the water splitting catalyst NiFeCoMoS/NFF, which is characterized by a 1D–2D–3D composite morphology. The NiFeCoMoS/NFF exhibited remarkable electrocatalytic performance with overpotentials of 123 for HER and 245 mV for OER at current densities 10 mA cm^−2^ while maintaining long-term stability. We attribute this level of electrocatalytic performance to an enhanced charge transfer process, an abundance of electrocatalytic active sites and synergistic effects at the heterojunction interfaces. Density functional theory calculations have demonstrated that the CoS/MoS interface facilitates more favorable H adsorption energetics while NiFeS accelerates the OER reactions, both features contributing to the overall electrolytic process. The double electrode that has been assembled incorporating NiFeCoMoS/NFF has generated current densities of 100 mA cm^−2^ at low voltages of 1.87 with good durability, establishing the effectiveness of this transition metal-based bifunctional electrocatalyst. The strategy adopted in this study, harnessing the multidimensional composite structure and heterojunction synergism, can serve as a template for developing novel electrocatalyst systems for efficient clean energy production.

## Data availability

The data that support the findings of this study are available from the corresponding author upon reasonable request.

## Conflicts of interest

There are no conflicts to declare.

## Supplementary Material

RA-015-D4RA08605H-s001
