# Supplementary material for: Multi-dimensional composite catalyst NiFeCoMoS/NFF for overall electrochemical water splitting
Source: RSC Adv. 2025 Feb 17;15(7):5305–15. doi: 10.1039/d4ra08605h (PMC11831736; doi:10.1039/d4ra08605h)
Supplement: RA-015-D4RA08605H-s001 [file RA-015-D4RA08605H-s001.pdf]

Supplementary Information (SI) for RSC Advances.
This journal is © The Royal Society of Chemistry 2025

Supporting Information

Zhaojun Tan[1*], Shuaihui Guo[1], Wen Wang[1], Gang Li[1], Zhenwei Yan[1*]

1. School of Mechanical Engineering, North China University of Water Resources and Electric Power, Zhengzhou 450045, PR China

*Corresponding author E-mail: 13603990078@163.com (Z. Tan), yanzhenwei@163.com (Z. Yan)

*Corresponding author Tel：+86 13603990078(Z. Tan), +86 18638513931(Z. Yan)

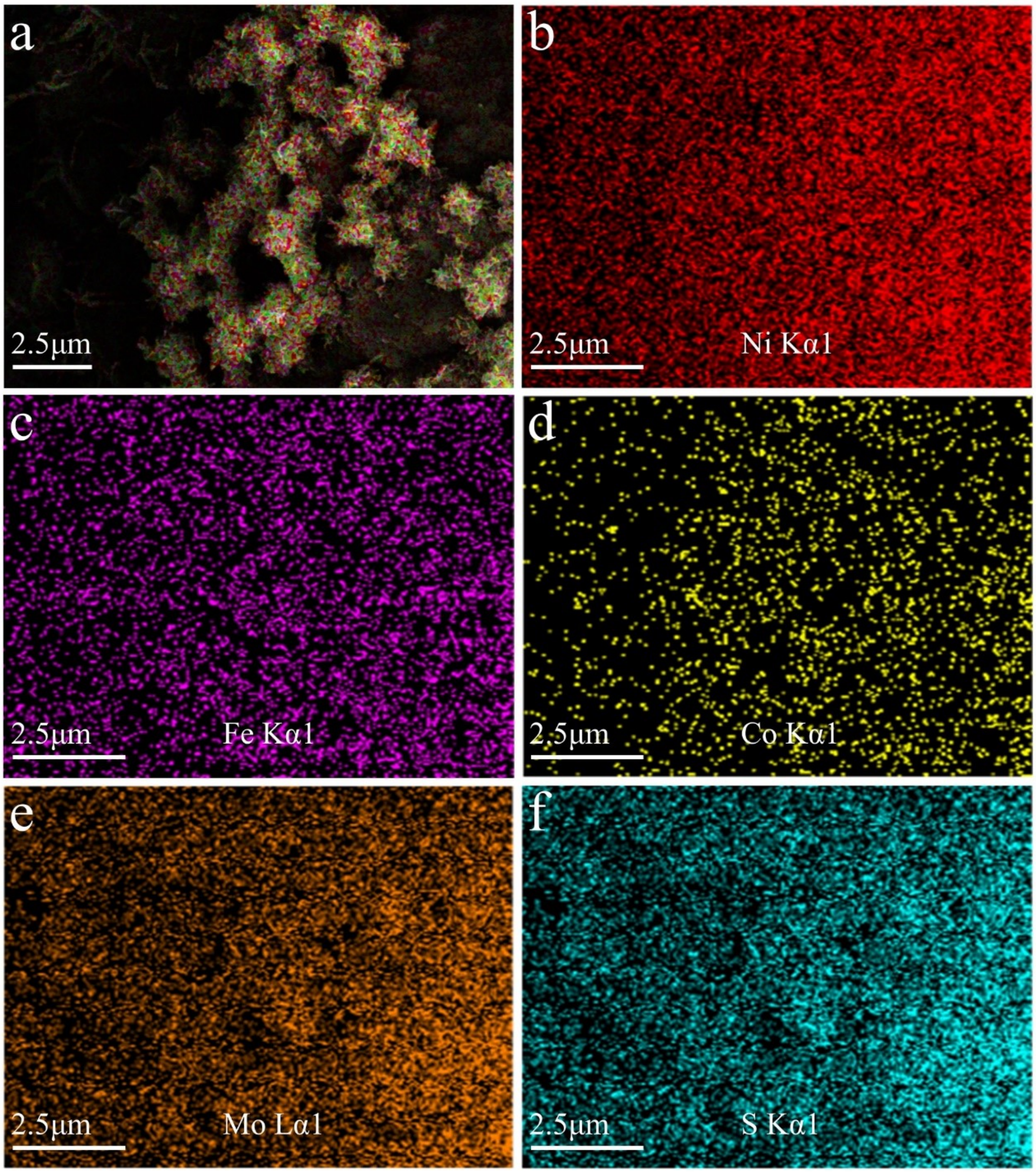

Figure 1 EDS mapping image of NiFeCoMoS/NFF

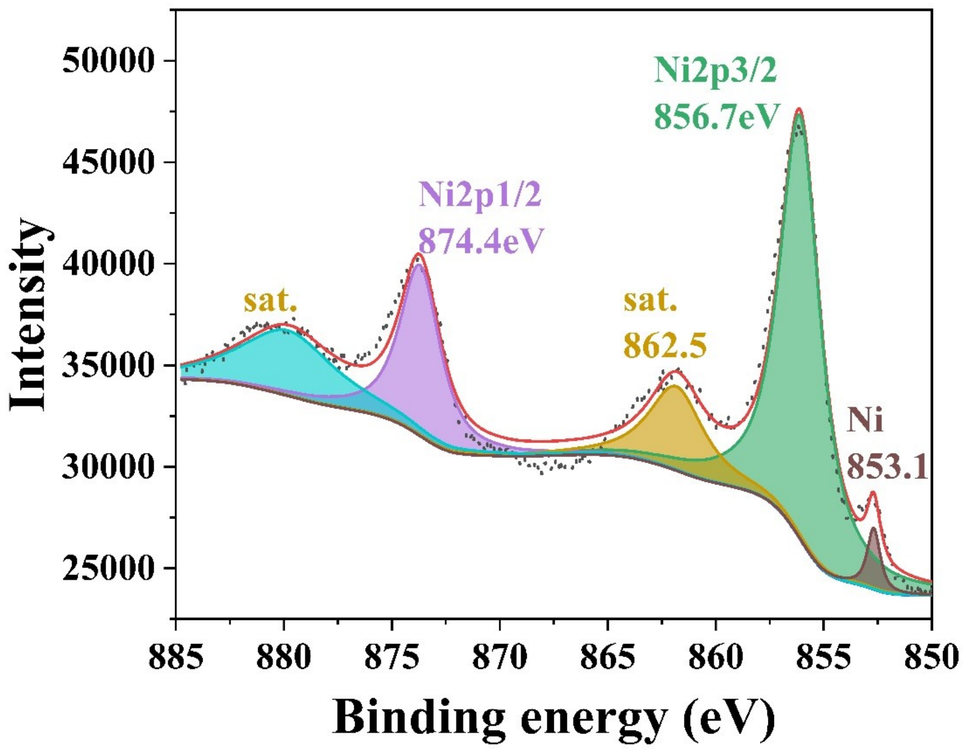

Figure 2 High-resolution XPS spectra for Ni 2p of Ni_3S_2

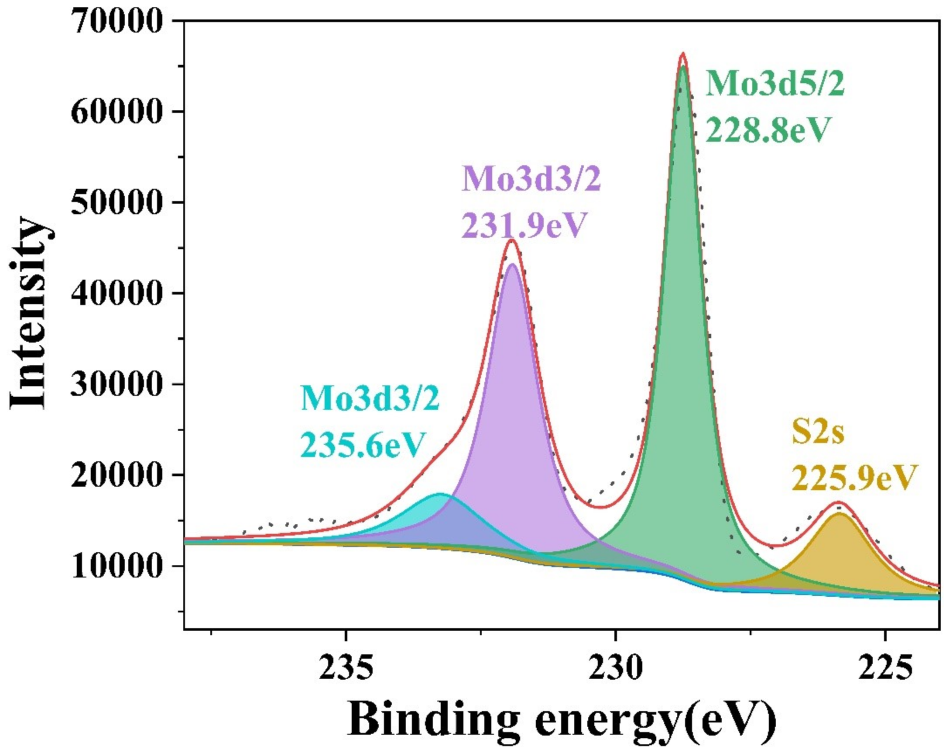

Figure 3 High-resolution XPS spectra for Mo 2p of MoS_2

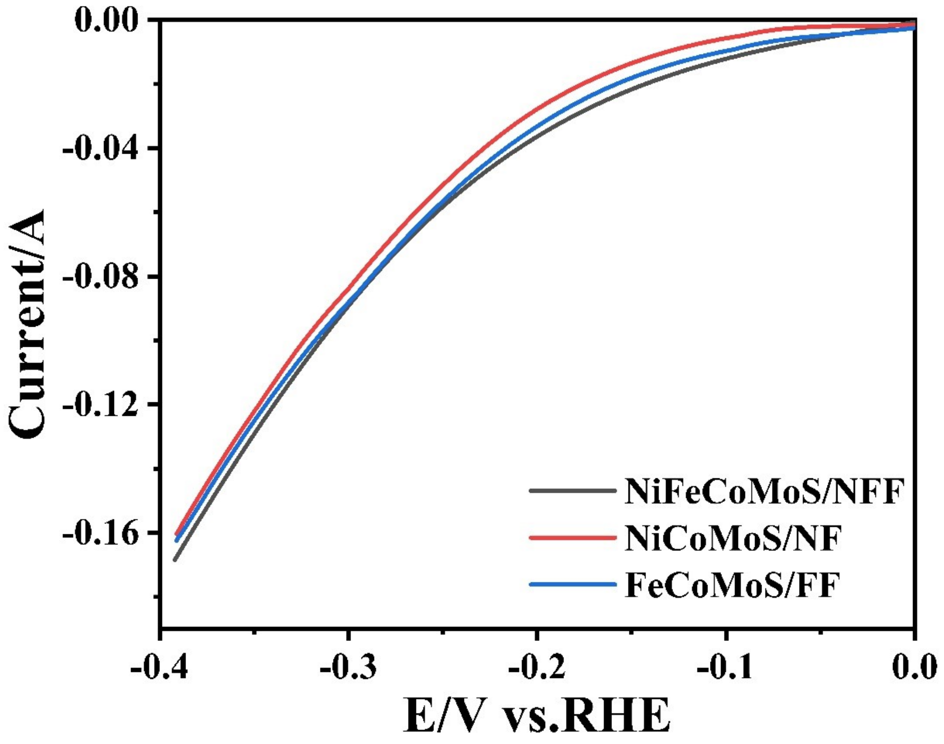

Figure 4 Polarization curves of HER

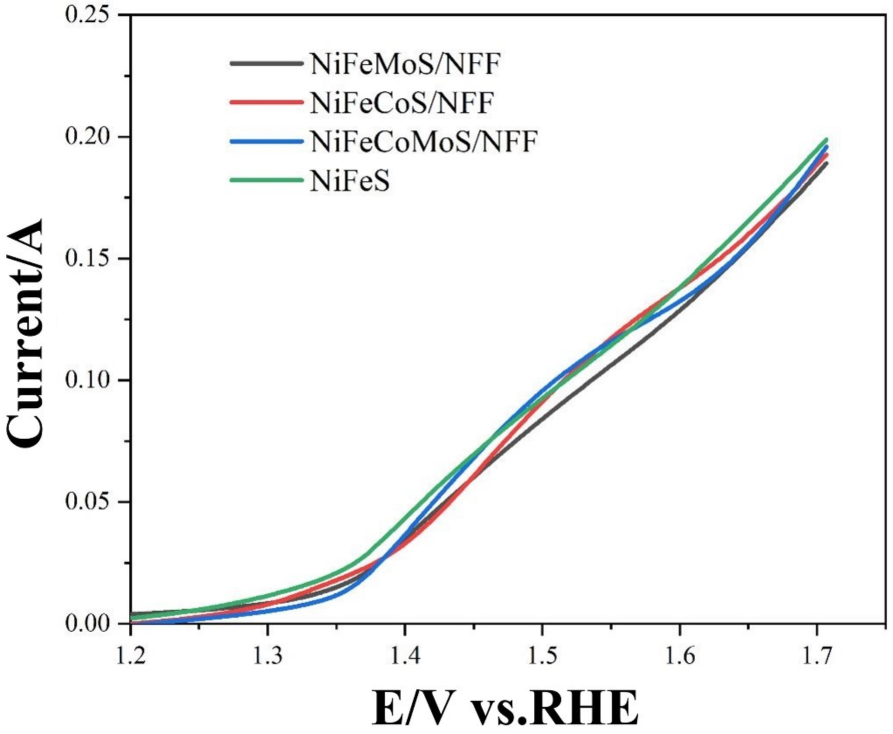

Figure 5 Polarization curves of OER

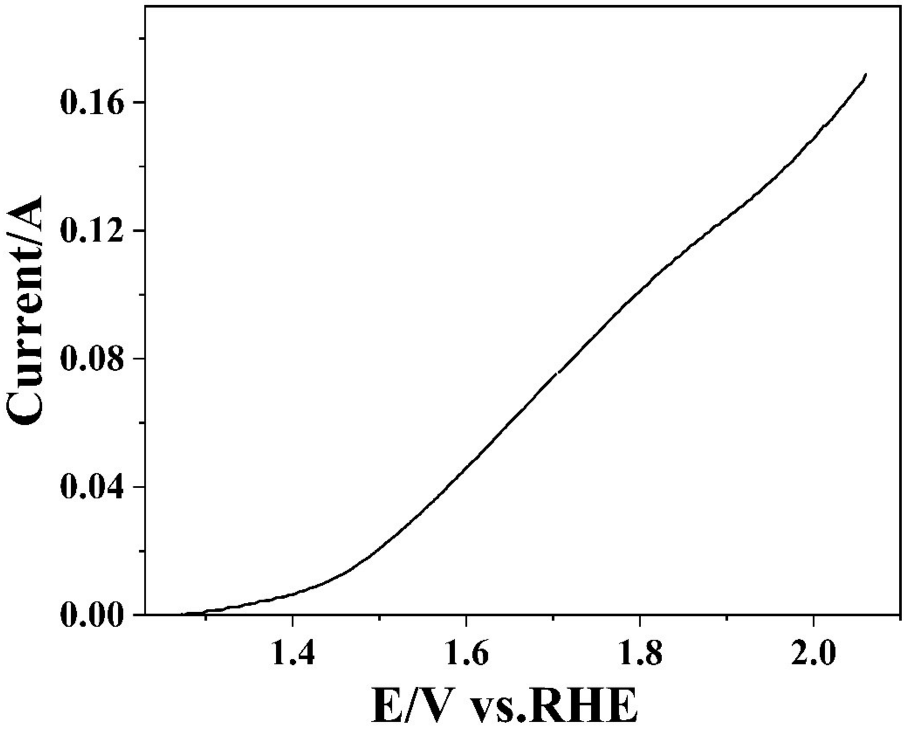

Figure 6 Polarization curve of two-electrode system

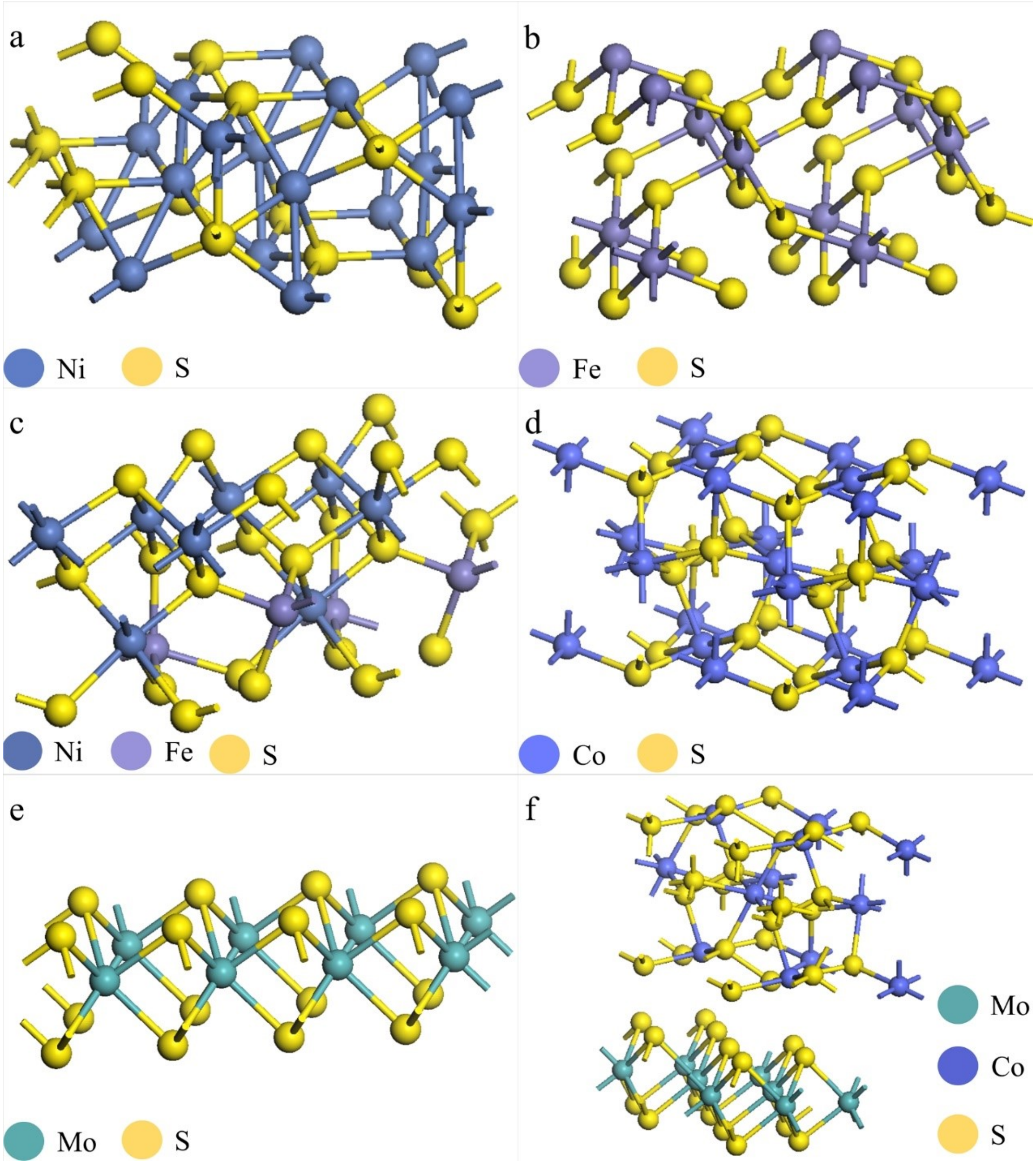

Figure 7 Chemisorption models of Ni_3S_2[1 0 1] (a), FeS_2[200] (b), Fe $(NiS_2)_2$ [1 1 1] (c), CoS_2 [2 0 0], MoS_2 and CoMoS.

Table1 Cdl and ECSA of HER

Catalysts	Cdl(mF.cm^{-2})	ECSA (cm^2)
CoMoS/FF	33.3	832.5
CoMoS/NF	29.9	747.5
NiFeCoMoS/NFF	32.2	805
NiFeMoS/NFF	12.4	310
NiFeCoS/NFF	15.3	382.5
NiFeS	21.2	530

Table2 Cdl and ECSA of OER

Catalysts	Cdl(mF.cm^{-2})	ECSA (cm^2)
CoMoS/FF	7.5	187.5
CoMoS/NF	21.3	532.5
NiFeCoMoS/NFF	31.9	797.5
NiFeMoS/NFF	30.6	765
NiFeCoS/NFF	32.5	812.5
NiFeS	32.7	817.5

Table3 Comparison of activity of the NiFeCoMoS/NFF with recently reported non-precious metal based electrocatalysts. The * indicates data was corrected by the iR loss.

Electrocatalyst	Electrolyte	η_{10} / mV	Substrate	Reference
Ni3S2-CoMoSx	1M KOH	1.52*	Ni foam	1
Co-Fe-NiSe2	1M KOH	1.52*	carbon cloth	2
MoS2/NiS2	1M KOH	1.59*	carbon cloth	3
MoS2/NiS	1M KOH	1.61	Ni foam	4
P- Co3O4	1M KOH	1.63	Ni foam	5
Ni/Mo2C-NCNF	1M KOH	1.64	carbon cloth	6
Fe-Ni@NC-CNTs	1M KOH	1.7	carbon cloth	7
NiCo/NiCoOx	1M KOH	1.72	Ni foam	8
δ-FeOOH	1M KOH	1.65	Ni foam	9
NiCo2S4	1M KOH	1.7	Ni foam	10

[1] Lingxue Zhao, Huaiyun Ge, Guanghui Zhang, et al. Hierarchical Ni3S2-CoMoSx on the nickel foam as an advanced electrocatalyst for overall water splitting. Electrochimica Acta,2021, 387 ,138538-138546.

[2] Yiqiang Sun, Kun Xu, Zengxi Wei, et al. Strong electronic interaction in dual-cation-incorporated NiSe2 nanosheets with lattice distortion for highly efficient overall water splitting. Adv. Mater.，2018，30, 1802121.

[3] Jinghuang Lin, Pengcheng Wang, Haohan Wang, et al. Defect-rich heterogeneous MoS2/NiS2 nanosheets electrocatalysts for efficient overall water splitting. Adv. Sci. 2019,6, 1900246.

[4] ZhangJie Zhai, Chao Li, Lei Zhang, et al. Dimensional construction and morphological tuning of heterogeneous MoS2/NiS electrocatalysts for efficient overall water splitting. J.Mater. Chem. A, 2018,6, 9833–9838.

[5] Zhichao Wang, Hongli Liu, Ruixiang Ge, et al. Phosphorus-doped Co3O4 nanowire array: a highly efficient bifunctional electrocatalyst for overall water splitting. ACS Catal. 2018,8,2236–2241.

[6] Meixuan Li, Yun Zhu, Huiyuan Wang, al. Ni strongly coupled with Mo2C encapsulated in nitrogen-doped carbon nanofibers as robust bifunctional catalyst for overall water splitting. Adv. Energy Mater. 2019, 9, 1803185.

[7]Xiaojia Zhao, Dr. Pradip Pachfule, Shuang Li, et al. Bifunctional electrocatalysts for overall water splitting from an

iron/nickel-based bimetallic metal-organic framework/dicyandiamide composite. Angew. Chem. Int. Ed. 2018,57, 8921–8926.
[8]Xiaodong Yan, KeXue Li, Lu Lyu, et al. From water oxidation to reduction: transformation from NixCo3-xO4 nanowires to NiCo/NiCoOx heterostructures. ACS Appl. Mater. Interfaces, 2016, 8, 3208–3214.
[9]Bin Liu, Yun Wang, Hui-Qing Peng ,et al. Iron vacancies induced bifunctionality in ultrathin feroxyhyte nanosheets for overall water splitting. Adv. Mater. ,2018,30, 1803144.
[10]Arumugam Sivanantham, Pandian Ganesan, Sangaraju Shanmugam. Hierarchical NiCo2S4 nanowire arrays supported on Ni foam: an efficient and durable bifunctional electrocatalyst for oxygen and hydrogen evolution reactions. Adv. Funct. Mater.,2016,26, 4661–4672.
